# Differentiating lung cancer and infection based on measurements of extracellular pH with acidoCEST MRI

**DOI:** 10.1038/s41598-019-49514-1

**Published:** 2019-09-10

**Authors:** Leila R. Lindeman, Kyle M. Jones, Rachel A. High, Christine M. Howison, Lisa F. Shubitz, Mark D. Pagel

**Affiliations:** 1 Cancer Biology Graduate Interdisciplinary Program, University of Arizona, Tucson, AZ USA; 2 Bioengineering Graduate Interdisciplinary Program, University of Arizona, Tucson, AZ USA; 3 Department of Medical Imaging, University of Arizona, Tucson, AZ USA; 4 Valley Fever Center for Excellence, University of Arizona, Tucson, AZ USA; 5 Department of Cancer Systems Imaging, MD Anderson Cancer Center, Houston, TX USA

**Keywords:** Cancer imaging, Diagnostic markers, Cancer imaging

## Abstract

Lung cancer diagnosis via imaging may be confounded by the presence of indolent infectious nodules in imaging studies. This issue is pervasive in the southwestern US where coccidioidomycosis (Valley Fever) is endemic. AcidoCEST MRI is a noninvasive imaging method that quantifies the extracellular pH (pHe) of tissues *in vivo*, allowing tumor acidosis to be used as a diagnostic biomarker. Using murine models of lung adenocarcinoma and coccidoidomycosis, we found that average lesion pHe differed significantly between tumors and granulomas. Our study shows that acidoCEST MRI is a promising tool for improving the specificity of lung cancer diagnosis.

## Introduction

According to 2015 estimates, lung cancer is the second most frequently diagnosed cancer and the leading cause of cancer-related mortality in men and women in the United States^1^. Early diagnosis can greatly improve prognosis for individuals with this disease^2^. When detected early, non-small cell lung cancer may be treated surgically and has a better prognosis relative to metastatic disease. Given the benefits of early detection, the United States Preventative Services Task Force (USPSTF) recently recommended annual low dose CT for lung cancer screening in individuals 55–80 years old with a history of smoking 30 packs per year, and individuals that quit smoking after age 40.

Unfortunately, while CT has adequate sensitivity to detect early lung cancer lesions, it is not specific to cancer diagnosis and also detects non-cancerous nodules^3^. One of the major disadvantages of the low-dose CT screening regimen is the high rate of false positives. Ninety-five percent of positive CTs are ultimately diagnosed as arising from conditions other than cancer^2^. Currently, the resolution of ambiguous nodules requires follow-up imaging studies or biopsy. Follow-up CTs that monitor lesion growth can be used to identify lung tumors vs. non-cancerous, size-stable nodules, but this longitudinal monitoring results in delayed diagnosis and treatment. Ambiguous nodules greater than 8 mm in diameter are typically referred for biopsy, an invasive procedure with inherent life-threatening risks. Ambiguous CT findings are common in the southwestern US, where early diagnosis of lung cancer is often confounded by the presence of granulomas from coccidioidomycosis (Valley Fever), which can appear morphologically identical to early-stage lung tumors via CT and conventional MRI. Approximately 150,000 people become infected with coccidioidomycosis annually, and about two-thirds of these infections never become clinically symptomatic^4^. Roughly 60% of these cases occur in southern Arizona, confounding the early detection of lung malignancies in this region^5^.

It is well established that tumors generate and export excess lactic acid due to upregulated glycolytic metabolism, resulting in strongly acidic extracellular pH (pHe) in the tumor microenvironment that is typically ≤7.1 pH units^6^. Normal tissue is typically pH neutral at 7.4 pH units^7^. *Coccidioides* fungi are known to excrete ammonia, likely resulting in alkaline pHe, >7.4 pH units, in infected tissues^8^. Infections can cause inflammation, and the inflammatory microenvironment can be mildly acidic (7.2–7.4 pH units). Therefore, an infectious nodule may be mildly acidic, pH-neutral, or mildly alkaline, and yet the nodule should not be strongly acidic^9^. Therefore, a strongly acidic pHe may be used to differentiate lung cancer from infection, which can accelerate diagnoses and avoid unnecessary biopsies.

We have developed a noninvasive imaging method that can measure tumor acidosis, known as acidoCEST MRI. This method is based on Chemical Exchange Saturation Transfer (CEST) MRI, which selectively saturates the magnetization of labile protons on exogenous contrast agents or endogenous biomolecules so that their coherent MR signal disappears, waits for these protons to exchange with protons in the bulk water pool, and then measures the resulting reduction in MRI signal from water. The chemical exchange rate of the protons between the saturated contrast agent and bulk water pool is dependent on pH, and therefore CEST MRI signals are pH dependent^10^.

AcidoCEST MRI uses the exogenous contrast agent, iopamidol, which is approved by the FDA for use with clinical CT^11^. Iopamidol has three labile amide protons that generate two CEST signals after saturation at MR frequencies 4.2 and 5.6 ppm. The exchange of amide protons with water is base-catalyzed, and the chemical exchange rates of these amide protons depend on pH in different ways. Therefore, an analysis of the CEST spectrum of iopamidol can measure pH independent of the concentration of the agent and MRI characteristics such as endogenous T_1_ relaxation time or CEST saturation efficiency^12,13^. AcidoCEST MRI has been previously applied to measure extracellular pH (pHe) in murine flank and orthotopic cancer xenograft models, and a murine lung fibrosis model^11,12,14–17^.

We sought to determine if acidoCEST MRI can distinguish between lung tumors and coccidioidomycosis granulomas by characterizing the pHe of those lesion types in preclinical mouse models. We selected a spontaneous, chemically induced, orthotopic model of murine adenocarcinoma, and a novel mutant model of coccidioidomycosis that is safe to handle in biosafety level 2 (BSL-2) facilities^18^. We compared average lesion pHe measurements between the two groups to determine the suitability of acidoCEST MRI for differentiating these types of lesions.

## Results

Our respiration-triggered acidoCEST-FISP MRI method successfully imaged both murine lung tumors and granulomas. Slow, steady breathing during this scanning procedure led to extended scan times relative to previous acidoCEST MRI studies. Despite this obstacle, we completed all scans without mouse fatality. The seven mice with spontaneous lung adenocarcinomas were very stable under anesthesia. Lung tumors took 17–46 weeks to reach a sufficient size for successful imaging, by which time mice were mature. The five mice infected with *Δcps1* were more fragile under anesthesia due to pulmonary inflammation and pneumonias that occurred after infection.

**Figure 1:**
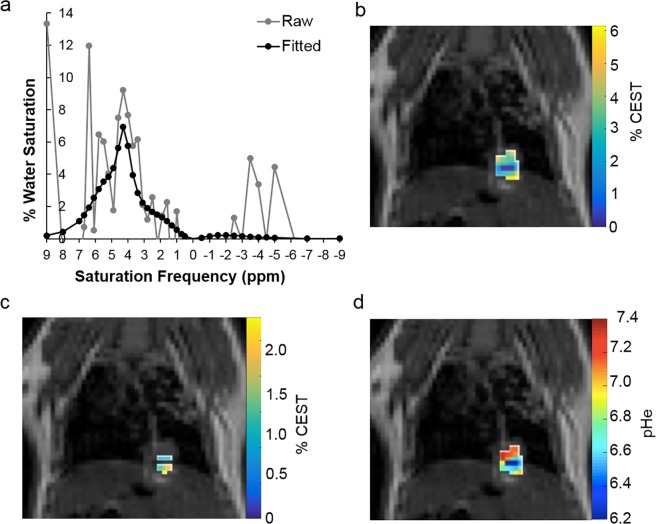
CEST effects from iopamidol are detected in lung tumors. (**a**) % CEST values were measured by selective saturation at each frequency. % CEST values were averaged and processed with Gaussian spatial smoothing, and then pre-injection results were subtracted from post-injection results to produce the experimental CEST spectrum in the graph. The Bloch-McConnell equations modified to include pH as a variable were used to iteratively fit a theoretical CEST spectrum to the experimental CEST spectrum. (**b**) Parametric map showing spatial CEST signal at 4.2 ppm. (**c**) Parametric map showing spatial CEST signal at 5.6 ppm. (**d**) Resulting spatial pHe map.

While pathology results from previous studies indicated that granulomas would appear within 2 weeks of infection, the key to successful *in vivo* imaging was to wait 3–4 weeks for the acute inflammatory background to clear after initial exposure to *Δcps1* spores^18^. When the acute inflammation cleared from the lungs, the mice had slow, steady breathing patterns. The data quality was sufficient to detect and fit CEST effects at 4.2 and 5.6 ppm in processed CEST spectra (Fig. 1a), and in spatial maps (Fig. 1b,c) resulting in a pHe map (Fig. 1d). At this 3–4 week time frame, the infectious nodules typically consist of small granulomas with a fibrogranulomatous mantle as a necrotic center of debris, containing degenerate and nondegenerate neutrophils, and occasional small empty spherules. Therefore, this stage of the infection is consistent with chronic, benign coccidioidomycosis that is more likely to confound lung tumor evaluations than acute coccidioidomycosis.

**Figure 2:**
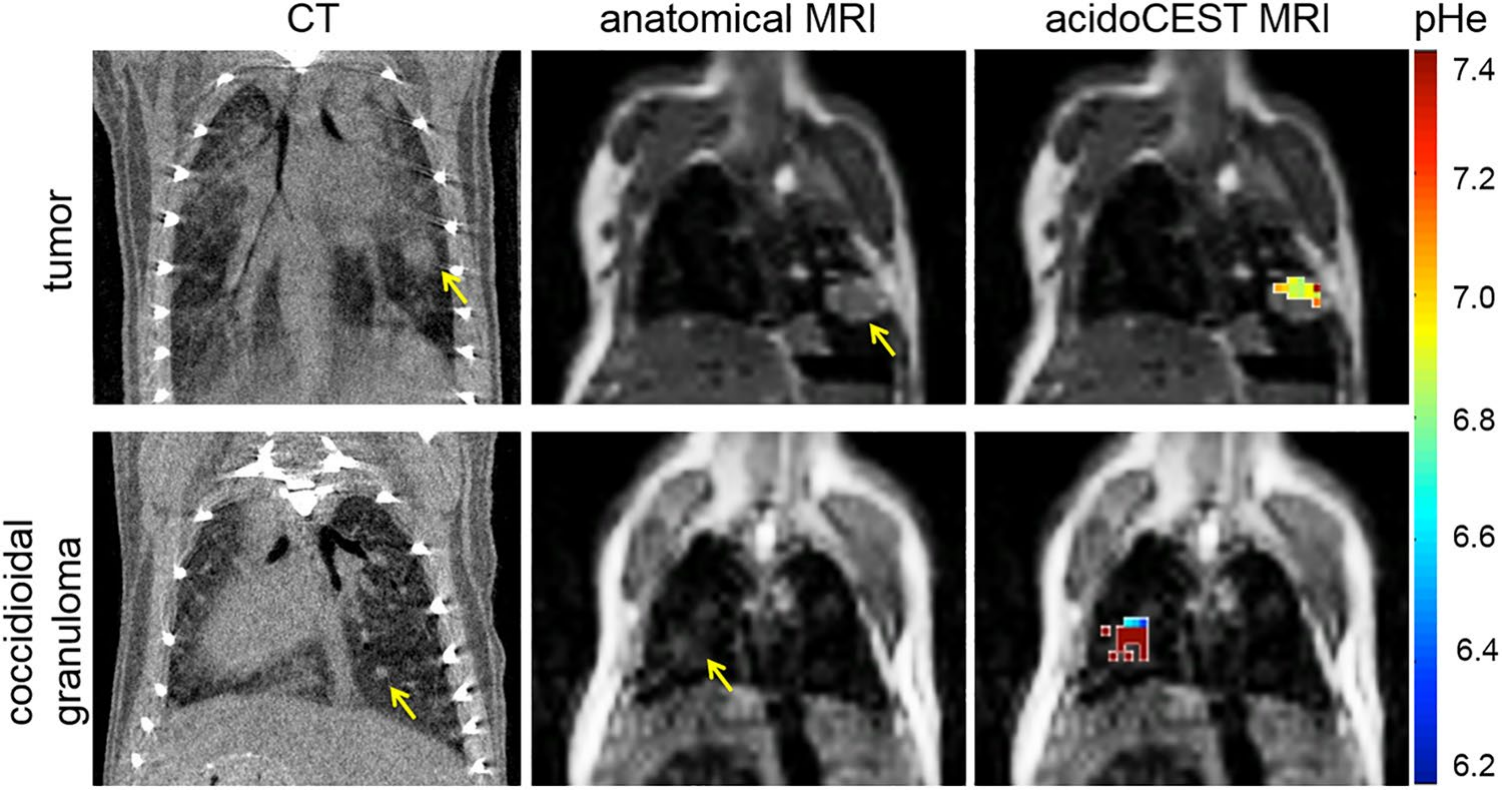
CT and MR images of lung tumors and infections. Murine lung adenocarcinomas and *Δcps1* coccidioidal granulomas present similarly as discrete gray masses in CT and anatomical MR images. AcidoCEST MRI differentiates clearly between the two lesions. Lesions appear in different locations in these images because these example CT and MR images were obtained with different mice.

CT and anatomical MRI are not able to clearly distinguish between granulomas and tumors, where both types of lesions appear as gray masses in the lung (Fig. 2a,b). This result in our mouse models parallels clinical observations in humans^3^. Conversely, our spatial pHe maps clearly differentiated between granulomas and tumors (Fig. 2c). These pHe maps showed acidosis in the lung tumors while the granulomas were only mildly acidic or pHe-neutral. We used user-selected regions of interest (ROI) to guide the quantification of lesion pHe during data analysis. We used manual ROI selection because acidoCEST MRI is not intended to improve the sensitivity of lung lesion detection, but rather to improve the specificity of diagnosis after a lesion has been detected.

**Figure 3:**
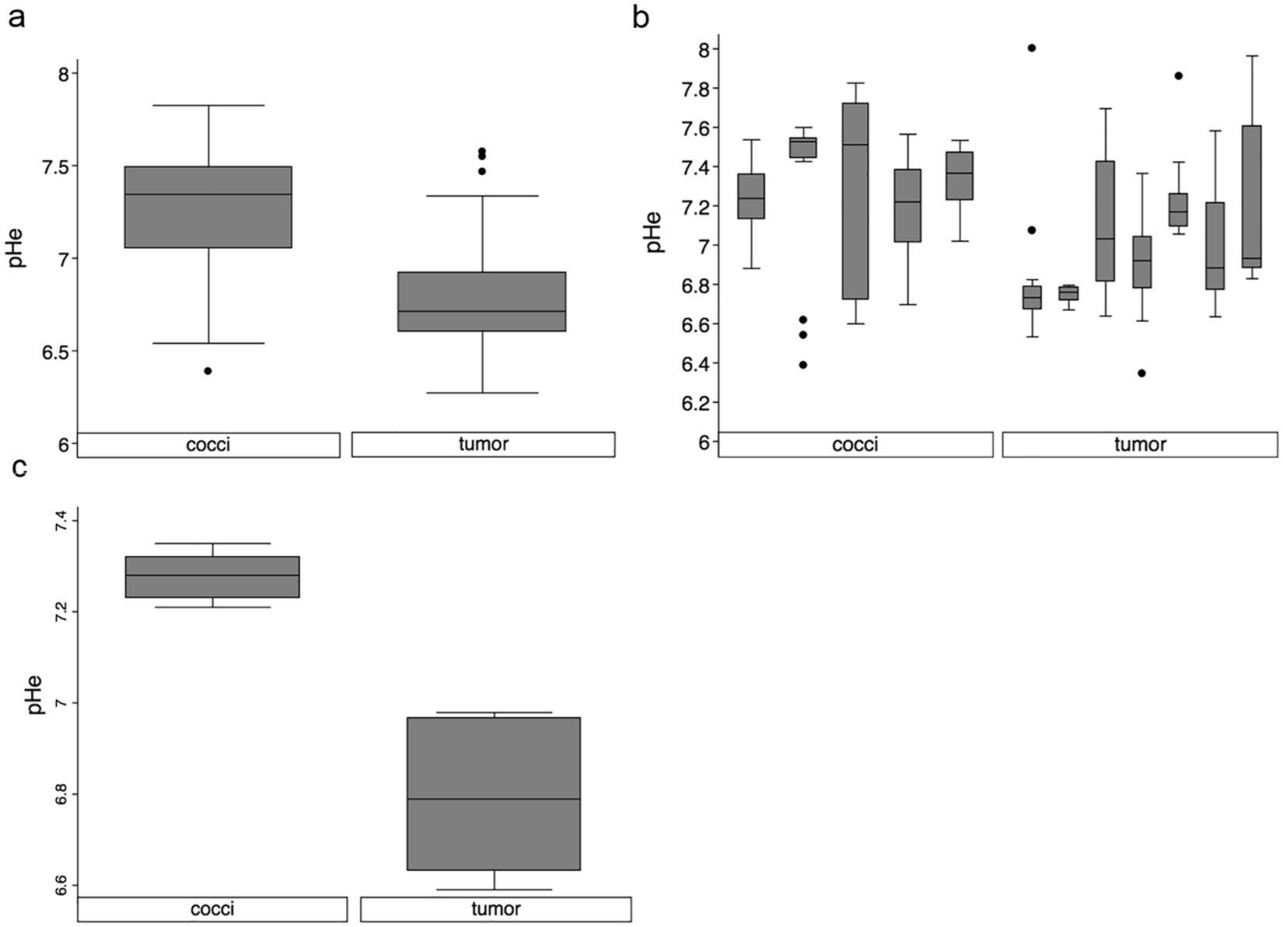
Average lesion pHe draws a clear distinction between neoplastic and coccidioidal groups. (**a**) A boxplot of pooled pixelwise pHe values in the tumor and cocci groups. (**b**) A boxplot of pixelwise pHe values for each individual mouse in the tumor and *Coccidioides* groups showed that the median lesion pHe values of seven mice with lung tumors were all lower than the median pHe values of five mice with coccidioidal granulomas. (**c**) A boxplot of median lesion pHe values in the neoplastic and coccidioidal groups (horizontal lines in the shaded boxes in panel b) shows that the distributions of these median pHe values were significantly different (*p* < 0.0001). All box plots show median or mean values as a horizontal line in the shaded box; the 25–50% interquartile and 50–75% interquartile ranges as a shaded box; the range of non-outliers as whiskers; and outliers as dots, where outliers are defined as values beyond an additional 1.5 interquartile ranges below and above the interquartile ranges.

We analyzed the distributions of pHe values in both types of lesions. The pHe values of all pixels from all tumors were pooled, and the same pooling was performed for all pixels of granulomas (Fig. 3a). These poolings showed a distinct difference between the median pHe in neoplastic and coccidioidal lesions, even though portions of the distributions overlapped and the two distributions were not statistically different. Pixelwise pHe distributions of individual mice in each group illustrated the variable spread of pHe values among animals in each group (Fig. 3b). The median pHe value for each mouse was determined from the pixelwise pHe values for each mouse (median values are shown as a horizontal line in each shaded box in Fig. 3b; these median values included all pixelwise pHe values including outliers that are shown as dots in Fig. 3b). These median pHe values for the granuloma group were all consistently higher than for the tumor group. The mean of these median pHe values were 7.28 (SD = 0.06) for the granulomas and 6.80 (SD = 0.15) for the tumors (the means are shown as a horizontal line in each shaded box in Fig. 3c). An unpaired, two-sided t-test confirmed that there was a statistically significant difference in the distribution of median pHe values between granulomas and tumors (*p* < 0.0001; these distributions are shown in Fig. 3c).

**Figure 4:**
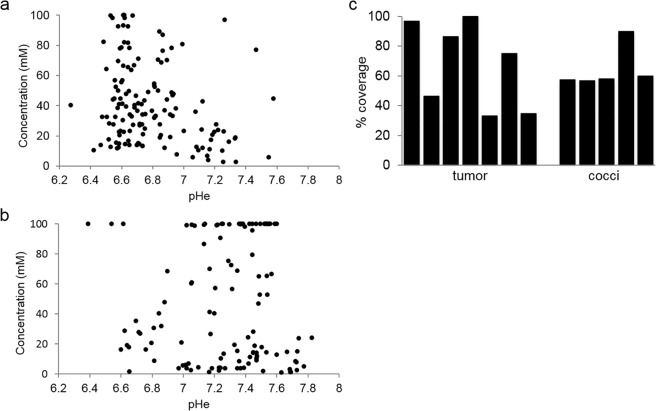
Iopamidol concentration was not correlated with pHe. (**a**) Scatterplot of iopamidol concentration (mM) versus pHe for lung tumors. (**b**) Scatterplot of iopamidol concentration (mM) versus pHe for *Δcps1* granulomas. (**c**) Percent coverage (number of quantifiable pixels/total size of ROI) for tumors and coccidioidal granulomas.

There was no relationship between iopamidol concentration and pHe value (Fig. 4a,b), which agreed with a previous study^16^. These scatterplots also illustrated that there was a wide distribution of iopamidol uptake values in both groups. The proportion of pixels where pHe could be quantified, relative to the total number of pixels within the ROI for the lesion, was expressed as a Percent Coverage for each individual mouse in the tumor and coccidioidal granuloma groups (Fig. 4c). Percent coverage was highly variable in the tumor group (range = 33.3–100.0%, mean = 67.5%, SD = 28.9%), whereas percent coverage was more consistent in the granuloma group (range = 56.7–90.0%, mean = 64.5%, SD = 14.3%). This result may reflect the well-known heterogeneity of solid tumors.

## Discussion

Our results indicate that the acidoCEST MRI method differentiates between lung tumors and coccidioidal granulomas. The acidic pHe values observed in lung tumors were consistent with tumor acidosis resulting from the Warburg effect^6^. We anticipated the neutral pHe values observed in coccidioidomycosis granulomas due to a combination of mild acidosis from inflammatory cells combined with the presence of ammonia in the extracellular space secreted by the *Coccidioides* organisms^8,9^. Notably, the deletion of the CPS1 gene in the *Δcps1* mutant model contains a DMAP1 regulatory domain and two AMP binding domains, and appears to encode for a protein that is part of a larger machinery involved in the synthesis of cell wall components. Therefore, the CPS1 deletion in our model does not directly affect ammonia-related metabolism. However, any gene mutation has potential to indirectly alter cell function, so that the *Δcps1* mutation may still affect the pHe of our infection model. Therefore, additional acidoCEST MRI studies with natural *Coccidioides* could be performed in future studies. In particular, our report justifies clinical translation of acidoCEST MRI to study the pHe of natural coccidioidomycosis.

More generally, additional studies with other models of lung infections, including infections that produce varying levels of ammonia and other metabolites, are warranted to expand the results of our initial studies. These additional studies can evaluate whether acidoCEST MRI can differentiate lung tumor from many types of lung infections, in addition to coccidioidomycosis. Ideally, these future studies should test infectious disease models with different mouse strains as performed in our current study, to ensure that the results are independent of mouse strain. Furthermore, future studies may investigate larger cohorts of mouse models of lung cancer and infectious diseases, to further investigate the range of pHe in each model.

Recent advances in MRI for preclinical models of lung tumors and infections are primarily concerned with increasing the anatomical sensitivity and accuracy of the modality to improve lesion detection and monitor lesion size. High-resolution, gated MRI sequences have been successfully applied to quantitatively monitor tumor growth over time in mouse models^19,20^. The threshold of detection in high-resolution MRI scans may be improved by employing an automated segmentation algorithm^21^. Alternatively, three-dimensional, ultrashort echo time (UTE) MRI has been performed with free-breathing animals, and the tumor burden results were shown to correlate well with *ex vivo* histology^22^. A similar free-breathing, UTE MRI approach has been used for the longitudinal, *in vivo* monitoring of aspergillosis infection in a preclinical mouse model^23^. The ability of UTE MRI to detect the presence of aspergillosis infection was equivalent to that of CT, and the MRI was more sensitive to gradual pathological changes in the lung. While these methods impact lung disease diagnosis by improving the sensitivity of imaging, they fail to improve the specificity by distinguishing lung tumors from infections.

More recently, a retrospective clinical study of unenhanced MRI signal intensity features from pulmonary lesions in a cohort of 29 patients with acute infections or tumors was able to differentiate between those two conditions with 95% specificity^24^. However, this study evaluated on-going, acute infections, requiring that diagnostic imaging be performed during the acute time frame. Our studies were performed after acute inflammation had cleared, eliminating the need for the imaging scan to be performed within a time frame relative to pathologic state. Our finding that extracellular pH can differentiate lung tumors from infections lays the foundation for the first non-invasive, differential diagnostic test for the discrimination of ambiguous lung lesions, and represents a major new application for molecular imaging of cancer.

## Methods

### Animal models

All mouse studies were conducted with the approval of the University of Arizona Institutional Animal Care and Use Committee in accordance with applicable guidelines and regulations. To produce a spontaneous, orthotopic model of lung adenocarcinoma, seven male A/J mice were treated with a single intraperitoneal injection of 1 mg/g of urethane in 0.2 mL PBS at 6 weeks of age. The lung adenocarcinomas reached a size of 1 mm at 25–26 weeks of age with average body weight of 29.4 g, which was suitable for MRI studies. Because *Coccidioides* spp. are biosafety level 3 aerosol pathogens, and also because the infection is rapidly fatal in mice before it can form stable granulomas, an avirulent (biosafety level 2) strain with the CPS1 gene removed, *Δcps1*^18^ was used to produce lung granulomas for imaging. Five female mice 5–17 weeks of age were infected via intranasal insufflation with 30 μL of *Δcps1* spores in 0.9% saline under ketamine (80 mg/kg of body weight)-xylazine (8 mg/kg) anesthesia as described previously^18^. BALB/c, C57BL/6j, and Swiss-Webster (SW) mice were given variable quantities (50,000–500,000 spores) of *Δcps1* either once, or twice 7 days apart. (Table 1) The mouse strains and dosages of Δ*cps1* were modified as studies proceeded to optimize the formation of granulomas and survival under anesthesia for imaging. Mice were imaged at 14, 21, or 28 days after the first exposure (Table 1). The average weight of the mice at the time of imaging varied by strain: Balb/c −19.7 g, C57BL/6j − 21.5 g, SW − 24.5 g.

**Table 1:** Δcps1 exposure times and doses by mouse strain.

Mouse strain	1st Exposure Age (weeks)	1st Exposure Dose	Booster	Booster Dose
Balb/c	17	500 K	no	n/a
C57BL/6 J	11	50 K	yes	50 K
SW group 1	9	100 K	yes	100 K
SW group 2	9	100 K	yes	100 K
SW group 3	5	50 K	yes	100 K

Prior to imaging, we anesthetized each mouse with 1.5–2.5% isofluorane in 1 L/min oxygen carrier gas. Although prolonged exposure to isoflurane anesthesia can cause mild acidosis throughout the mouse body, previous evidence suggests that this acidosis lowers pHe by only ~0.05 units during a 30–40 minute scan^25^. Furthermore, this mild acidosis may affect both the lung tumors and infectious nodules, so that an isoflurane-induced difference in pHe may be less than 0.05 units for these lesion types. For these reasons, the use of isoflurane was justified in our studies. A 27-gauge tail vein catheter was used to deliver iopamidol contrast agent^15^. We used a fiber optic rectal probe to monitor temperature, and a respiration pad to monitor respiration rate (SA Instruments, Inc., Stony Brook, NY). To facilitate optimal respiratory gating, anesthesia levels were maintained such that mice had a respiratory rate of 20–50 breaths per minute. Mouse body temperature was maintained at 36.5–37.5 °C using warm air.

### Image acquisition

We performed anatomical MRI scans with each mouse to locate the tumor or granuloma using parameters listed in Table 2. AcidoCEST MRI was performed by applying selective saturation at 3.5 μT power, followed by a Fast Imaging with Steady-state Precession (FISP) acquisition^26^. We acquired images to produce four CEST spectra for each image pixel prior to i.v. administration of the contrast agent. Then a 200 μL bolus of 370 mg/ml (976 mM) of iopamidol (Isovue®, Bracco Diagnostics, Inc.) was injected i.v. within 60 sec, and the injection line was connected to a syringe pump that infused 400 μL/hour of agent during the next 30–40 minutes. Six sets of CEST MR images were acquired immediately after injection to produce six post-injection CEST spectra for each imaging pixel^15^.

**Table 2:** Parameters for MRI scans.

Scan Type	Anatomical MRI	acidoCEST MRI
Acquisition method	RARE MRI	CEST-FISP MRI
TR	1075.57 ms	3.7 ms
TE	12.7 ms	1.6 ms
Excitation flip angle	90.0°	15°
In-plane spatial resolution	0.0453 cm/pixel	0.0453 cm/pixel
Matrix size	128 × 128	128 × 128
FOV (cm)	5.8 × 5.8	5.8 × 5.8
Slice thickness	1 mm	2 mm
Number of slices	5	1
RARE factor	1	n/a
Number of experiments	1	4 pre-, 6 post-
Number of averages	1	1
Saturation power	n/a	3.5 μT
Number of saturation frequencies	n/a	40^a^
Saturation Time	n/a	600 ms
Total acquisition time	2 min 17.0 sec	1 min 21.6 sec

^a^−3300 to −900 Hz in 600 Hz increments; −750 to 750 Hz in 150 Hz increments;

810 to 2700 Hz in 90 Hz increments; 3000 Hz, and 3300 Hz.

For respiration-gated acidoCEST MRI, we achieved steady-state saturation by applying 10 square-shaped pulses for 600 ms each (a total of 6 s). After this pulse train, the protocol checked the respiration trigger. An active trigger would cue MR signal collection to commence^13^. An inactive trigger led to the application of another 600 ms saturation pulse followed by another trigger check. This process was repeated until the protocol detected an active trigger and acquired the MR signal to produce an image.

CT scans were performed with a Siemens Inveon μCT instrument using 220° rotation, 440 projections, and a 100 ms settle time. Images were processed with 2 × 2 binning to produce 36 μm effective pixel size, with a 36 mm transaxial field of view and 45 mm axial field of view. Exposures were set at 50 kV voltage and 500 μA current, with a 400 ms exposure time. CT reconstruction used a Feldkamp algorithm, without downsampling, slight noise reduction, a Shepp-Logan filter, and no beam-hardening correction.

### Image analysis

To analyze acidoCEST MRI results, we averaged the four pre-injection images at each saturation frequency and applied a Gaussian spatial smoothing algorithm to improve the signal-to-noise ratio^15^. We performed the same steps to process the six post-injection images. The resulting pre-injection image was subtracted from the post-injection image at each saturation frequency to eliminate CEST signals from static endogenous sources. Pixels with contrast below 2√2(scan noise) were discarded from the analysis^27^. CEST spectra were then obtained for each imaging pixel in the tumor or granuloma. CEST spectra were fit with the Bloch-McConnell equations (the Bloch equations that include chemical exchange) modified to directly include pH as a fitting parameter, as previously described^13,28^. This analysis method also estimated the concentration of the agent (capped at 100 mM), endogenous T1 and T2 relaxation time constants, B1 saturation power, and B0 value. Pixels with pHe values below 6.2 pH units were excluded, based on an analysis of reliability previously evaluated with phantoms^15,28^. We capped the upper range of permitted pHe values at 7.9 to allow for the possibility of alkaline pHe in the granuloma microenvironment that could be caused by the presence of extracellular ammonia^8^. Median values of lesion pHe for each mouse were determined rather than mean values to mitigate the influence of a few pixels with extremely high or low pHe values (shown as dots in Fig. 3b). Unpaired, two-sided t-tests were used to compare median pHe values between tumor and *Coccidioides* groups. Statistical calculations were performed in STATA 13.1.

## Data Availability

The datasets generated during and/or analyzed during the current study are available from the corresponding author on reasonable request.
